# Anhydrous polymeric zinc(II) penta­noate

**DOI:** 10.1107/S1600536808008283

**Published:** 2008-06-07

**Authors:** Richard A. Taylor, Henry A. Ellis

**Affiliations:** a Department of Chemistry, University of the West Indies, Mona, Kingston 7, Jamaica

## Abstract

The structure of the title compound, poly[di-μ-penta­noato-zinc(II)], [Zn{CH_3_(CH_2_)_3_COO}_2_]_*n*_, consists of a three-dimensional polymeric layered network with sheets parallel to the (100) plane, in which tetra­hedrally coordinated zinc(II) ions are connected by penta­noate bridges in a *syn*–*anti* arrangement. The hydro­carbon chains are in the fully extended all-*trans* conformation and are arranged in a tail-to-tail double bilayer.

## Related literature

For related literature, see: Clegg *et al.* (1986 ▶); Blair *et al.* (1993 ▶); Dumbleton & Lomer (1965 ▶); Glover (1981 ▶); Goldschmied *et al.* (1977 ▶); Ishioka *et al.* (1998 ▶); Lacouture *et al.* (2000 ▶); Lewis & Lomer (1969 ▶); Lomer & Perera (1974 ▶); Peultier *et al.* (1999 ▶); Segedin *et al.* (1999 ▶).
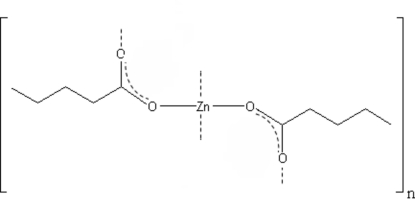


## Experimental

### 

#### Crystal data

[Zn(C_5_H_9_O_2_)_2_]
*M* *_r_* = 267.63
Monoclinic, 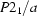

*a* = 9.389 (2) Å
*b* = 4.7820 (10) Å
*c* = 29.126 (7) Å
β = 104.256 (7)°
*V* = 1267.5 (5) Å^3^
*Z* = 4
Mo *K*α radiation
μ = 1.93 mm^−1^
*T* = 293 (2) K
0.30 × 0.30 × 0.05 mm

#### Data collection

Rigaku R-AXIS IIC image-plate diffractometer
Absorption correction: multi-scan (*CrystalClear*; Rigaku, 2000 ▶) *T*_min_ = 0.621, *T*_max_ = 1.000 (expected range = 0.564–0.908)
7493 measured reflections
2125 independent reflections
1965 reflections with *I* > 2σ(*I*)
*R*_int_ = 0.061

#### Refinement

*R*[*F* ^2^ > 2σ(*F* ^2^)] = 0.062
*wR*(*F* ^2^) = 0.126
*S* = 1.17
2125 reflections
138 parameters
H-atom parameters constrained
Δρ_max_ = 0.32 e Å^−3^
Δρ_min_ = −0.52 e Å^−3^

### 

Data collection: *CrystalClear* (Rigaku, 2000 ▶); cell refinement: *CrystalClear*; data reduction: *CrystalClear*; program(s) used to solve structure: *SIR92* (Altomare *et al.*, 1994 ▶); program(s) used to refine structure: *SHELXL97* (Sheldrick, 2008 ▶); molecular graphics: *Mercury* (Macrae *et al.*, 2006 ▶) and *DIAMOND* (Bergerhoff *et al.*, 1996 ▶); software used to prepare material for publication: *SHELXL97*.

## Supplementary Material

Crystal structure: contains datablocks global, I. DOI: 10.1107/S1600536808008283/cf2188sup1.cif

Structure factors: contains datablocks I. DOI: 10.1107/S1600536808008283/cf2188Isup2.hkl

Additional supplementary materials:  crystallographic information; 3D view; checkCIF report

## Figures and Tables

**Table:** 

Zn1—O1	1.950 (3)
Zn1—O3	1.966 (3)
Zn1—O2^i^	1.947 (3)
Zn1—O4^ii^	1.963 (4)

**Table:** 

O2^i^—Zn1—O1	107.80 (15)
O2^i^—Zn1—O4^ii^	112.66 (15)
O1—Zn1—O4^ii^	116.62 (17)
O2^i^—Zn1—O3	113.19 (15)
O1—Zn1—O3	100.89 (15)
O4^ii^—Zn1—O3	105.21 (14)

Symmetry codes: (i) 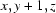; (ii) 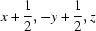.

**Fig. 1.**
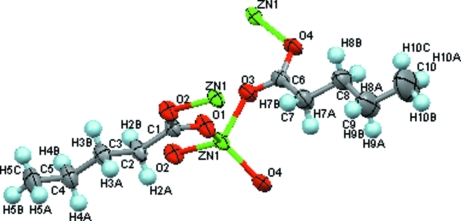
: Asymmetric unit of zinc(II) n-pentanoate: Displacement ellipsoids are drawn at the 75% probability level.

**Fig. 2.**
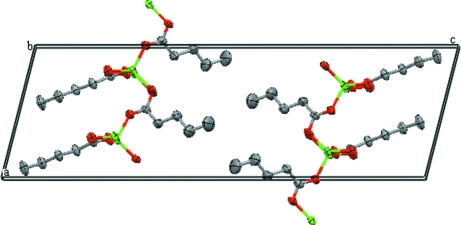
: Projection down the b axis. Displacement ellipsoids are drawn at the 50% probability level.

**Fig. 3.**
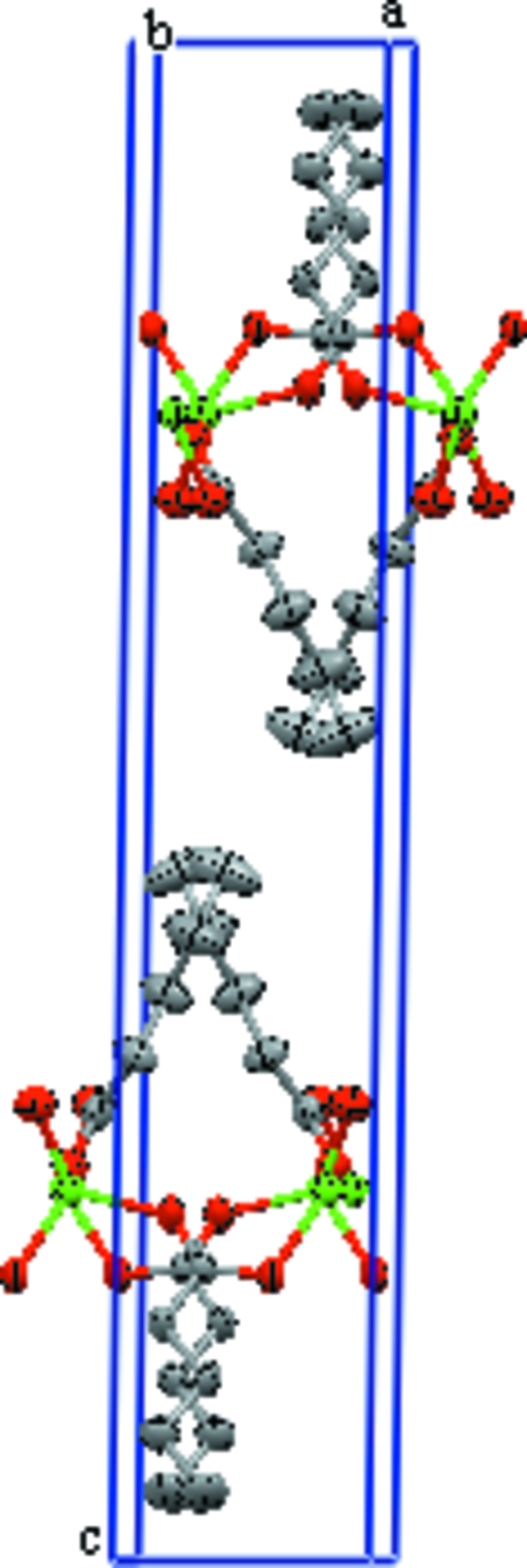
: View down the a axis (hydrogen atoms omitted). Displacement ellipsoids are drawn at the 50% probability level.

**Fig. 4.**
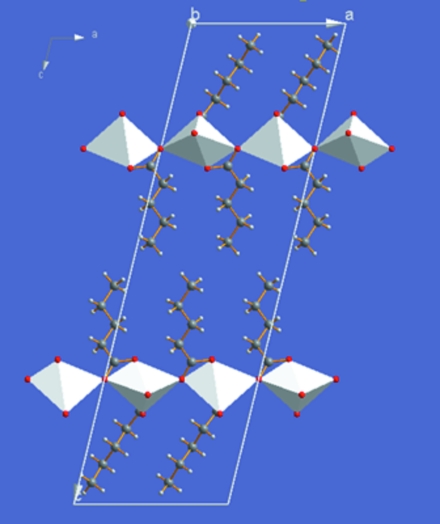
: Unit-cell contents, showing alternating tetrahedra of oxygen atoms around zinc ions in the zinc basal planes.

## supplementary crystallographic information

### Comment

Long-chain metal carboxylates do not easily form crystals suitable for single-crystal X-ray analysis; usually, the crystals are thin needles that are fragile and, in many cases exhibit micro-twinning. Consequently, the few structures that have been reported are those of the short-chain homologues (Dumbleton & Lomer, 1965; Lewis & Lomer, 1969; Glover, 1981; Lomer & Perera, 1974; Ishioka *et al.*, 1998). For the zinc(II) series those reported include anhydrous zinc(II) acetate (Clegg *et al.*, 1986), propionate (Goldschmied *et al.*, 1977), butanoate (Blair *et al.*, 1993), hexanoate and heptanoate (Segedin *et al.*, 1999; Peultier *et al.*, 1999) and octanoate (Lacouture *et al.*, 2000). The compounds are isostructural in the sense that the zinc ions have a tetrahedral geometry of oxygen atoms and are bridged by bidentate ligands. In this study, anhydrous zinc(II) pentanoate, (I), was investigated in order to elucidate its crystal structure.

The structure (Fig. 1) is four-coordinate, where each zinc ion is tetrahedrally coordinated by oxygen atoms from four different pentanoate ligands. The four pentanoate ligands around zinc are of the *Z*,*E*-type bridging bidentate mode; that is, they are bonded in a *syn*-anti arrangement to two tetrahedral zinc ions. Geometric data indicate that the Zn—O bond lengths are not equivalent and clearly point to unsymmetrical bonding around the zinc ion.

The alkyl chains of the pentanoate groups are in the fully extended all-*trans* conformation. There is excellent agreement of the C—C bond lengths and C—C—C angles with published values for hydrocarbon chains in a fully extended all-*trans* conformation (Lomer & Perera, 1974). There are four formula units in the unit cell and two distinct basal planes, resulting in a double bilayer lamella arrangement forming a polymeric network (Fig. 2) with an alternating packing of the hydrocarbon chains in neighbouring bilayers. When viewed down the *b* axis, the hydrocarbon chains, which are tilted with respect to the zinc basal planes, are in each bilayer aligned in different planes. The structure appears very different when viewed down the *a* axis (Fig. 3), where in one bilayer the chains appear to zigzag and cross at the bonds along the C—C axis. In the other bilayer the chains are tilted towards each other and appear to cross each other at carbon atom number 4.

The molecular packing (Fig. 4) highlights the distorted tetrahedra around the zinc ions. In one basal plane, the vertices of the tetrahedra alternate parallel and perpendicular to the vertical plane throughout and in the other basal plane the vertices alternate at the top and bottom throughout. This arrangement allows for alternating basal planes in the overall structure to be identical.

There is interaction between parallel sheets through bidentate bridging, resulting in a three-dimensional sheet-like/layered polymeric network where the chains are arranged tail-to-tail, arising from van der Waals interactions in sheets parallel to the *ac* plane.

### Experimental

Single crystals of zinc(II) pentanoate were prepared from the reaction of zinc oxide (0.407 g) and n-pentanoic acid (5.0 cm^3^; >100% excess) in approximately 100 cm^3^ of ethanol. The white suspension was refluxed until the solution was transparent. The resulting hot, colorless solution was filtered by suction and the filtrate left to cool to room temperature. After about six days, long, thin, colourless, plate-like single crystals, some in clusters, crystallized from solution. The crystals were then removed, air-dried, and kept in sealed vials at ambient temperature.

### Refinement

H atoms were positioned geometrically and refined as riding, with C—H = 0.97 Å and *U*_iso_(H) = 1.2*U*_eq_(C) for methylene, and C—H = 0.96 Å and *U*_iso_(H) = 1.5*U*_eq_(C) for methyl groups. The crystal was weakly diffracting at high angles.

### Crystal data

**Table:**

[Zn(C_5_H_9_O_2_)_2_]	*D*_x_ = 1.402 Mg m^−^^3^
*M**_r_* = 267.63	Melting point: 425.5 K
Monoclinic, *P*2_1_/*a*	Mo *K*α radiation λ = 0.71073 Å
*a* = 9.389 (2) Å	Cell parameters from 7493 reflections
*b* = 4.7820 (10) Å	θ = 2.2–25.0º
*c* = 29.126 (7) Å	µ = 1.93 mm^−^^1^
β = 104.256 (7)º	*T* = 293 (2) K
*V* = 1267.5 (5) Å^3^	Thin block, colourless
*Z* = 4	0.30 × 0.30 × 0.05 mm
*F*_000_ = 560	

### Data collection

**Table:**

Rigaku R-AXIS IIC image-plate diffractometer	2125 independent reflections
Radiation source: rotating-anode X-ray tube	1965 reflections with *I* > 2σ(*I*)
Monochromator: graphite	*R*_int_ = 0.062
Detector resolution: 105 pixels mm^-1^	θ_max_ = 25.0º
*T* = 100(2) K	θ_min_ = 2.2º
φ scans	*h* = −11→11
Absorption correction: multi-scan(CrystalClear; Rigaku, 2000)	*k* = −5→5
*T*_min_ = 0.621, *T*_max_ = 1.000	*l* = −34→34
7493 measured reflections	

### Refinement

**Table:**

Refinement on *F*^2^	Secondary atom site location: difference Fourier map
Least-squares matrix: full	Hydrogen site location: inferred from neighbouring sites
*R*[*F*^2^ > 2σ(*F*^2^)] = 0.062	H-atom parameters constrained
*wR*(*F*^2^) = 0.126	*w* = 1/[σ^2^(*F*_o_^2^) + (0.0408*P*)^2^ + 3.0707*P*] where *P* = (*F*_o_^2^ + 2*F*_c_^2^)/3
*S* = 1.17	(Δ/σ)_max_ < 0.001
2125 reflections	Δρ_max_ = 0.32 e Å^−^^3^
138 parameters	Δρ_min_ = −0.52 e Å^−^^3^
Primary atom site location: structure-invariant direct methods	Extinction correction: none

### Special details

**Table:**

Geometry. All e.s.d.'s (except the e.s.d. in the dihedral angle between two l.s. planes) are estimated using the full covariance matrix. The cell e.s.d.'s are taken into account individually in the estimation of e.s.d.'s in distances, angles and torsion angles; correlations between e.s.d.'s in cell parameters are only used when they are defined by crystal symmetry. An approximate (isotropic) treatment of cell e.s.d.'s is used for estimating e.s.d.'s involving l.s. planes.
Refinement. Refinement of *F*^2^ against ALL reflections. The weighted *R*-factor *wR* and goodness of fit *S* are based on *F*^2^, conventional *R*-factors *R* are based on *F*, with *F* set to zero for negative *F*^2^. The threshold expression of *F*^2^ > σ(*F*^2^) is used only for calculating *R*-factors(gt) *etc*. and is not relevant to the choice of reflections for refinement. *R*-factors based on *F*^2^ are statistically about twice as large as those based on *F*, and *R*- factors based on ALL data will be even larger.

### Fractional atomic coordinates and isotropic or equivalent isotropic displacement parameters (Å^2^)

**Table:**

	*x*	*y*	*z*	*U*_iso_*/*U*_eq_	
C1	0.7184 (5)	−0.3109 (10)	0.19302 (17)	0.0350 (10)	
C2	0.7780 (6)	−0.1602 (11)	0.15666 (19)	0.0456 (13)	
H2A	0.8636	−0.0544	0.1731	0.055*	
H2B	0.7047	−0.0267	0.1407	0.055*	
C3	0.8215 (6)	−0.3404 (11)	0.11919 (19)	0.0462 (13)	
H3A	0.8985	−0.4689	0.1346	0.055*	
H3B	0.7374	−0.4503	0.1029	0.055*	
C4	0.8750 (7)	−0.1677 (13)	0.0835 (2)	0.0571 (15)	
H4A	0.9595	−0.0591	0.1000	0.069*	
H4B	0.7983	−0.0376	0.0686	0.069*	
C5	0.9177 (9)	−0.3437 (17)	0.0453 (2)	0.081 (2)	
H5A	0.9946	−0.4712	0.0599	0.122*	
H5B	0.9517	−0.2233	0.0239	0.122*	
H5C	0.8338	−0.4473	0.0282	0.122*	
C6	0.4620 (5)	0.1354 (11)	0.29619 (18)	0.0405 (12)	
C7	0.5718 (6)	−0.0210 (14)	0.3329 (2)	0.0569 (16)	
H7A	0.6541	0.1024	0.3456	0.068*	
H7B	0.6085	−0.1756	0.3176	0.068*	
C8	0.5177 (7)	−0.1364 (17)	0.3739 (2)	0.0666 (18)	
H8A	0.4695	0.0125	0.3870	0.080*	
H8B	0.4450	−0.2800	0.3621	0.080*	
C9	0.6363 (9)	−0.258 (2)	0.4128 (3)	0.097 (3)	
H9A	0.7112	−0.1168	0.4237	0.116*	
H9B	0.6817	−0.4123	0.4001	0.116*	
C10	0.5825 (11)	−0.362 (3)	0.4546 (3)	0.139 (4)	
H10A	0.5363	−0.2109	0.4672	0.208*	
H10B	0.6642	−0.4303	0.4787	0.208*	
H10C	0.5128	−0.5099	0.4446	0.208*	
O1	0.6932 (4)	−0.1838 (7)	0.22800 (13)	0.0486 (9)	
O2	0.6954 (4)	−0.5724 (7)	0.18803 (12)	0.0438 (9)	
O3	0.4976 (4)	0.2344 (7)	0.26038 (12)	0.0431 (8)	
O4	0.3333 (4)	0.1625 (8)	0.30100 (13)	0.0480 (9)	
Zn1	0.68833 (6)	0.21140 (11)	0.24407 (2)	0.0358 (2)	

### Atomic displacement parameters (Å^2^)

**Table:**

	*U*^11^	*U*^22^	*U*^33^	*U*^12^	*U*^13^	*U*^23^
C1	0.040 (3)	0.031 (3)	0.038 (3)	0.000 (2)	0.017 (2)	0.001 (2)
C2	0.065 (4)	0.032 (3)	0.049 (3)	−0.004 (2)	0.032 (3)	0.000 (2)
C3	0.061 (4)	0.035 (3)	0.049 (3)	0.001 (2)	0.027 (3)	−0.006 (2)
C4	0.071 (4)	0.058 (4)	0.050 (3)	0.000 (3)	0.031 (3)	0.001 (3)
C5	0.110 (6)	0.092 (6)	0.059 (4)	−0.004 (5)	0.052 (4)	−0.008 (4)
C6	0.038 (3)	0.037 (3)	0.051 (3)	−0.001 (2)	0.020 (2)	−0.004 (2)
C7	0.045 (3)	0.077 (4)	0.051 (3)	0.011 (3)	0.017 (3)	0.018 (3)
C8	0.051 (4)	0.093 (5)	0.057 (4)	−0.003 (3)	0.017 (3)	0.022 (4)
C9	0.076 (5)	0.144 (9)	0.070 (5)	0.010 (5)	0.015 (4)	0.047 (5)
C10	0.121 (9)	0.214 (13)	0.081 (6)	0.009 (8)	0.025 (6)	0.073 (7)
O1	0.073 (3)	0.0289 (18)	0.055 (2)	−0.0028 (17)	0.037 (2)	−0.0019 (16)
O2	0.058 (2)	0.0271 (18)	0.051 (2)	−0.0039 (15)	0.0227 (18)	0.0000 (16)
O3	0.040 (2)	0.047 (2)	0.0457 (19)	0.0031 (15)	0.0176 (16)	0.0053 (16)
O4	0.036 (2)	0.064 (3)	0.050 (2)	0.0026 (17)	0.0219 (17)	0.0016 (18)
Zn1	0.0426 (4)	0.0295 (3)	0.0410 (3)	−0.0012 (2)	0.0210 (2)	−0.0022 (3)

### Geometric parameters (Å, °)

**Table:**

C1—O1	1.258 (6)	C7—C8	1.512 (8)
C1—O2	1.271 (6)	C7—H7A	0.970
C1—C2	1.498 (6)	C7—H7B	0.970
C2—C3	1.523 (7)	C8—C9	1.496 (9)
C2—H2A	0.970	C8—H8A	0.970
C2—H2B	0.970	C8—H8B	0.970
C3—C4	1.506 (7)	C9—C10	1.515 (10)
C3—H3A	0.970	C9—H9A	0.970
C3—H3B	0.970	C9—H9B	0.970
C4—C5	1.525 (8)	C10—H10A	0.960
C4—H4A	0.970	C10—H10B	0.960
C4—H4B	0.970	C10—H10C	0.960
C5—H5A	0.960	Zn1—O1	1.950 (3)
C5—H5B	0.960	O2—Zn1^i^	1.947 (3)
C5—H5C	0.960	Zn1—O3	1.966 (3)
C6—O4	1.256 (6)	O4—Zn1^ii^	1.963 (4)
C6—O3	1.263 (6)	Zn1—O2^iii^	1.947 (3)
C6—C7	1.491 (7)	Zn1—O4^iv^	1.963 (4)
			
O1—C1—O2	120.5 (4)	C8—C7—H7A	108.2
O1—C1—C2	121.2 (4)	C6—C7—H7B	108.2
O2—C1—C2	118.4 (4)	C8—C7—H7B	108.2
C1—C2—C3	116.5 (4)	H7A—C7—H7B	107.4
C1—C2—H2A	108.2	C9—C8—C7	114.0 (6)
C3—C2—H2A	108.2	C9—C8—H8A	108.8
C1—C2—H2B	108.2	C7—C8—H8A	108.8
C3—C2—H2B	108.2	C9—C8—H8B	108.8
H2A—C2—H2B	107.3	C7—C8—H8B	108.8
C4—C3—C2	112.2 (4)	H8A—C8—H8B	107.7
C4—C3—H3A	109.2	C8—C9—C10	113.7 (7)
C2—C3—H3A	109.2	C8—C9—H9A	108.8
C4—C3—H3B	109.2	C10—C9—H9A	108.8
C2—C3—H3B	109.2	C8—C9—H9B	108.8
H3A—C3—H3B	107.9	C10—C9—H9B	108.8
C3—C4—C5	113.1 (5)	H9A—C9—H9B	107.7
C3—C4—H4A	109.0	C9—C10—H10A	109.5
C5—C4—H4A	109.0	C9—C10—H10B	109.5
C3—C4—H4B	109.0	H10A—C10—H10B	109.5
C5—C4—H4B	109.0	C9—C10—H10C	109.5
H4A—C4—H4B	107.8	H10A—C10—H10C	109.5
C4—C5—H5A	109.5	H10B—C10—H10C	109.5
C4—C5—H5B	109.5	C1—O1—Zn1	133.1 (3)
H5A—C5—H5B	109.5	C1—O2—Zn1^i^	117.8 (3)
C4—C5—H5C	109.5	C6—O3—Zn1	128.3 (3)
H5A—C5—H5C	109.5	C6—O4—Zn1^ii^	115.0 (3)
H5B—C5—H5C	109.5	O2^iii^—Zn1—O1	107.80 (15)
O4—C6—O3	120.7 (5)	O2^iii^—Zn1—O4^iv^	112.66 (15)
O4—C6—C7	119.0 (5)	O1—Zn1—O4^iv^	116.62 (17)
O3—C6—C7	120.3 (4)	O2^iii^—Zn1—O3	113.19 (15)
C6—C7—C8	116.2 (5)	O1—Zn1—O3	100.89 (15)
C6—C7—H7A	108.2	O4^iv^—Zn1—O3	105.21 (14)

Symmetry codes: (i) *x*, *y*−1, *z*; (ii) *x*−1/2, −*y*+1/2, *z*; (iii) *x*, *y*+1, *z*; (iv) *x*+1/2, −*y*+1/2, *z*.
